# Biochar-Dual Oxidant Composite Particles Alleviate the Oxidative Stress of Phenolic Acid on Tomato Seed Germination

**DOI:** 10.3390/antiox12040910

**Published:** 2023-04-11

**Authors:** Yuting Tu, Jinchun Shen, Zhiping Peng, Yanggui Xu, Zhuxian Li, Jianyi Liang, Qiufang Wei, Hongbo Zhao, Jichuan Huang

**Affiliations:** 1 Institute of Agricultural Resources and Environment, Guangdong Academy of Agricultural Sciences, Guangzhou 510640, China; tuyuting@gdaas.cn (Y.T.);; 2 College of Horticulture, South China Agricultural University, Guangzhou 510642, China; 3 Key Laboratory of Plant Nutrition and Fertilizer in South Region, Ministry of Agriculture, Guangzhou 510640, China; 4 Guangdong Key Laboratory of Nutrient Cycling and Farmland Conservation, Guangzhou 510640, China; 5 College of Natural Resources and Environment, South China Agricultural University, Guangzhou 510642, China

**Keywords:** *p*-Coumaric acid, tomato seed germination, biochar, calcium peroxide, persulfate, ROS, metabolome, transcriptome

## Abstract

Phenolic acid is a well-known allelochemical, but also a pollutant in soil and water impeding crop production. Biochar is a multifunctional material widely used to mitigate the phenolic acids allelopathic effect. However, phenolic acid absorbed by biochar can still be released. In order to improve the removal efficiency of phenolic acids by biochar, the biochar-dual oxidant (BDO) composite particles were synthesized in this study, and the underlying mechanism of the BDO particles in ameliorating *p*-coumaric acid (*p*-CA) oxidative damage to tomato seed germination was revealed. Upon *p*-CA treatment, the BDO composite particles application increased the radical length, radical surface area, and germination index by 95.0%, 52.8%, and 114.6%, respectively. Compared to using biochar or oxidants alone, the BDO particles addition resulted in a higher removal rate of *p*-CA and produced more O_2_^•−^, HO^•^, SO_4_^•−^ and ^1^O_2_ radicals via autocatalytic action, suggesting that BDO particles removed phenolic acid by both adsorption and free radical oxidation. The addition of BDO particles maintained the levels of the antioxidant enzyme activity close to the control, and reduced the malondialdehyde and H_2_O_2_ by 49.7% and 49.5%, compared to the *p*-CA treatment. Integrative metabolomic and transcriptomic analyses revealed that 14 key metabolites and 62 genes were involved in phenylalanine and linoleic acid metabolism, which increased dramatically under *p*-CA stress but down-regulated with the addition of BDO particles. This study proved that the use of BDO composite particles could alleviate the oxidative stress of phenolic acid on tomato seeds. The findings will provide unprecedented insights into the application and mechanism of such composite particles as continuous cropping soil conditioners.

## 1. Introduction

Plants produced allelochemicals, which are secondary metabolites that mainly consist of phenolic compounds, terpenoids, long chain fatty acids, and nitrogen-containing compounds [1,2]. Among them, phenolic acids, including *p*-coumaric acid, *p*-hydroxybenzoic acid, coumaric acid, benzoic acid, ferulic acid, cinnamic acid, and others, have diverse functions and a high relative abundance in soil [3]. According to previous studies, phenolic acid induced allelopathic stress has negative effects on plants and their interactions with the environment [4,5].

Allelopathic effects of phenolic acids have been observed in over 50 plant species, such as tomato, soybean, strawberry, and panax ginseng [6,7]. A high concentration of phenolic acids can affect a cascade of physiological and biochemical processes in plants, such as causing oxidative stress induced by the production of excessive reactive oxygen species (ROS), regulating the metabolism of plant hormones and the expression of related genes, changing membrane permeability, altering root structure, limiting nutrient absorption, influencing photosynthesis and respiration, and ultimately inhibiting plant growth [8,9]. For *Cassia sophera* L., phenolic acids inhibit seedling growth with a decrease in dry biomass and morphological changes in the contraction of epidermal cells [10]. Wu et al. discovered that the concentration of benzoic acid was proportional to the degree of inhibition on ginseng root hair development, as well as the concentrations of proline and malondialdehyde (MDA) in root tissue [11]. Notably, phenolic acids suppress the expression of genes involved in photosynthesis, redox, and ion transport, including chlorophyll-binding protein (CBP), peroxidase (POD), superoxide dismutase (SOD), and ion channel proteins (ICP) [12]. In addition, phenolic acid stress induces the expression of several stress-responsive genes, such as the *GST* (Glutathione S-transferase) gene family, the *GR* (glutathione reductase) gene family, ATP-binding cassette (ABC) transporters, and transcription factors (*WRKY*, *MYB*, and *AP2/ERF*). [13,14]. Furthermore, the overabundance of phenolic acids in soil frequently results in a disaster in rhizosphere micro-ecology, such as an imbalance in rhizosphere microbial diversity, a drop in soil pH, and the suppression of soil enzyme activity [15].

Biochar is a multifunctional material generated from the high-temperature carbonization of biomass, which possesses abundant porosity and a high absorption capacity [16]. Several studies have been conducted in recent years to investigate the use of biochar to mitigate the allelopathic effect of phenolic acids on plant growth. Wang et al. demonstrated that biochar can promote the growth of *Malus hupehensis* Rehd. seedlings under phenolic acid stress, improve photosynthetic parameters and antioxidant enzyme activity and reduce the accumulation of MDA, reactive oxygen species (O_2_^•−^ and H_2_O_2_) [17]. However, other studies have shown that the highly concentrated phenolic acids absorbed by biochar inhibit the growth of plant roots [18]. In addition, the phenolic acids absorbed by biochar may release back into the cultivation medium through desorption [19]. In order to eliminate phenolic acid more completely, a chemical oxidant was considered to be coupled with biochar to improve the removal effect of phenolic acid.

Calcium peroxide (CP) and persulfate (PS) are the most widely employed solid oxidants for eliminating refractory organics. Significant stability against liquid H_2_O_2_ is demonstrated by CP, slowly releasing H_2_O_2_ (Equation (1)) and prolonging the reaction time. The produced H_2_O_2_ is then degraded further into hydroxyl radicals (HO^•^, E_0_ = 2.8 V) by activation, such as ferrous ions (Equation (2)) [20]. PS can create sulfate radicals (SO_4_^•−^, E_0_ = 2.6 V) when activated by transition metals (Equation (3)), heat, or UV radiation [21]. Several studies over the last two decades have demonstrated that these oxidants may rapidly decompose organic compounds containing benzene rings, such as polycyclic aromatic hydrocarbons (PAHs), polychlorinated biphenyls (PCBs), and methyl-butyl ether (MTBE) [22,23]. Wang et al. recently discovered that the dual peroxide oxidant system with CP and PS was more effective than either CP or PS alone [24].

CaO_2(s)_ + 2H_2_O → H_2_O_2_ +Ca(OH)_2(s)_ (1)

H_2_O_2_ + Fe^2+^ →HO^•^ + OH^−^ + Fe^3+^ (2)

S_2_O_8_^2−^ + Fe^2+^ →SO_4_^•−^ + SO_4_^2−^ + Fe^3+^ (3)

To the best of our knowledge, there is limited evidence on the impact of exogenously applied biochar and CP/PS systems on allelochemical elimination, and the microscopic regulation mechanism is also unknown. Therefore, biochar, CP, and PS were employed in the present study to prepare biochar-dual oxidant composite particles (BDO particles). The tomato plant was chosen as the experimental crop because it is one of the nightshade crops with the largest growing area and is susceptible to phenolic acid allelopathy. Due to the high soil concentration and considerable allelopathic effect on tomatoes, *p*-coumaric acid (*p*-CA) was used as a model allelochemical [25]. In this research, we studied the effect of BDO composite particles on the germination rate and antioxidant enzyme activity of tomato seeds under *p*-CA stress. The removal efficiency of *p*-CA from novel BDO particles was also investigated. We further explored the biological mechanism of BDO composite particles mitigating the allelopathic oxidative stress of *p*-CA on tomato seed germination with combined transcriptomic and metabolomic analyses.

## 2. Materials and Methods

### 2.1. Materials

Analytical grade calcium peroxide (CP), potassium persulfate (PS), *p*-coumaric acid (*p*-CA), formic acid, arboxymethyl cellulose, attapulgite (>98% purity), and high-performance liquid chromatography-grade methanol were purchased from Shanghai Macklin Biochemical Co., Ltd. (Shanghai, China). Biochar (BC) was prepared by pyrolyzing coconut husk at 650 °C for two hours (heating rate: 20 °C/min) in a horizontal furnace with flowing nitrogen. The main production process of biochar-dual oxidant composite particles (BDO particles) was grinding and sifting the BC, CP, PS, attapulgite, and arboxymethyl cellulose through a 200-mesh sieve, and then mixing the powders at a mass ratio of 100:11:39:4:2. After that, the appropriate amount of water was added to prepare spherical particles with a diameter of 2 mm. The composite particles were rapidly blast-dried at 40 °C. For comparison, biochar particles (BCp), calcium peroxide particles (CPp), and potassium persulfate particles (PSp) were also produced using the same procedure in this study. Seeds of tomato (*Solanum lycopersicum* L. cv. Red-Arrow) were obtained from the Guangdong Academy of Agricultural Sciences. The Petri dishes, filter papers, tweezers, pipettes, and distilled water were typically sterilized in a steam autoclave for 30 min before being used in seed germination experiments.

### 2.2. Seed Germination Experimental Design

During the seed germination experiments, tomato seeds were first sterilized in 5% sodium hypochlorite for 10 min, rinsed with sterilized distilled water (ddH_2_O) three times, and then immersed for 6 h at 25 °C in darkness. Following that, 50 tomato seeds were placed in each 9 cm-diameter Petri dish with two layers of filter paper. In each treatment, 10 mL of sterilized distilled water or *p*-CA solution was applied to the Petri plates, with or without particle addition. The seeds were subsequently cultured in a growth chamber maintained at 25 ± 1 °C, 75 ± 2% relative humidity. The light intensity of the cool white fluorescent lamps and photoperiod were set at around 9000 lux and a 16 h light/8 h dark cycle, respectively. Each Petri plate was gently shaken three times each day to facilitate the transfer of treatment fluid. Every two days, 2 mL of ddH_2_O was added to Petri dishes in the ultra-clean workbench to maintain consistent hydration for the germinating seeds until data collection was complete.

To determine the optimal amounts of phenolic acid stress for tomato seed germination, a screening test was carried out using 10 mL *p*-CA with various concentrations (0, 200, 400, 600, and 800 mg/L). Each treatment was replicated four times. The germination potential, germination rate, and radical length were assessed. The screening results (Table S1) revealed that the germination rate and root length of tomato seeds were reduced significantly when the *p*-CA concentration exceeded 400 mg/L. Therefore, a concentration of 400 mg/L *p*-CA was used as the simulated phenolic acid stress concentration. Subsequently, six treatments were designed to investigate the alleviate effects of BDO particles on tomato seed germination under phenolic acid stress, including ddH_2_O (CK), *p*-CA stress treatment (*p*-CA), *p*-CA + 0.0250 g BC particles (*p*-CA + BC_P_), *p*-CA + 0.0027 g CP particles (*p*-CA + CP_P_), *p*-CA + 0.0098 g PS particles (*p*-CA + PS_P_), *p*-CA + 0.0375 g BDO particles (*p*-CA + BOD_P_). The *p*-CA concentration was fixed at 400 mg/L, and the total solid addition of BC, CP, and PS therapies was 0.0375 g, equivalent to the dosage of BDO particles. The experiment was set up using a completely randomized block design. We used four biological replicates for each treatment. Each replicate consisted of 10 Petri dishes with 50 tomato seeds on each Petri dish.

### 2.3. Analysis of Seed Germination Indices

The whole germination test lasted for 10 days, and the number of germinated seeds was recorded each day. In each treatment, four dishes were randomly selected from each group for evaluation of seed germination indicators. Tomato seeds were considered germinated when the radical length exceeded 2 mm. The germination potential (GP) and germination rate (GR) were calculated as the proportion of germinated seeds to the total number of seeds used after 3 and 7 days of treatment (%), respectively. Radical length (RL) and radical surface area (RSA) were assessed for 7-day-old seedlings using Epson Expression 12000XL scanners equipped with WinRHIZO Pro 2021 software (Regent Instruments Inc., Quebec, Canada). The germination index (GI) was calculated by the following equation (Equation (4)):

GI = (number of germinated seeds in treatment × radical length in treatment) × 100%/(number of germinated seeds in control × radical length in control)(4)

To examine the allelopathic effects of different treatments on tomato seed germination, the allelopathy response index (RI) was evaluated. When the treatment value (T) was equal to or greater than the control group value (C), the RI was calculated by RI = 1 − C/T. The RI was defined as RI = T/C − 1 if T was smaller than C. The synthetical allelopathic effect index (SE) was derived by calculating the arithmetic mean of the RI of all test indices in the same treatment [26].

### 2.4. Physicochemical Characteristics of the Liquid Extracts and Free Radicals

The fluid in the Petri plate was collected after 1, 2, 3, 5, and 7 days of germination to evaluate residual *p*-CA concentrations. A certain amount of ddH_2_O was added to the Petri dish to maintain the same weight as at the beginning of the experiment. The extracted liquid was then filtered through 0.22 μm Millipore membrane filters. The *p*-CA concentration in the filtrate was measured using a Waters Alliance e2695 HPLC system equipped with a Waters 2489 UV/visible detector (Waters Co., Milford, MA, USA) and a C18 column (5.0 m, 4.6 mm 250 mm). The detection wavelength was adjusted at 288 nm. The mobile phase was a 70:30 volume mixture of methanol and water containing 0.1% formic acid, with a flow rate of 0.6 mL/min. The free radicals such as sulfate radical (SO_4_^•−^), hydroxyl radical (HO^•^), superoxide radical (O_2_^•−^), and singlet oxygen (^1^O_2_) were detected using electron paramagnetic resonance (EPR) on A300-10-12 spectrometer (Bruker BioSpin GmbH, Rheinstetten, Germany) with 0.1 mol/L DMPO and TEMP as spintrapping agents.

### 2.5. Analysis of Lipid Peroxidation and Antioxidant Enzyme Activities

Hydrogen peroxide (H_2_O_2_) and MDA were used as biomarkers of membrane lipid peroxidation caused by oxidative stress [27]. The absorbance of a hydroperoxide-titanium complex at 415 nm was used to ascertain the amount of H_2_O_2_ in the seed radical [28]. MDA levels and antioxidant enzyme activities such as superoxide dismutase (SOD), peroxidase (POD), and catalase (CAT) were measured using the Assay Kit (Sangon Biotech Co., Ltd., Shanghai, China). In summary, a 0.3 g radical sample was collected and transferred to a mortar before being homogenized in an ice bath with 3 mL of phosphoric acid buffer (pH = 7.8). The mixture was then centrifuged at 4 °C for 10 min (10,000 r/min). The obtained supernatants were analyzed using the corresponding determination kits. The MDA concentration was measured at wavelengths of 600, 532, and 450 nm.

### 2.6. Metabolomic Analysis

In this study, tomato seeds were grown for 7 days in CK, *p*-CA, and *p*-CA + BDOp treatments and were collected and washed three times with ddH_2_O. Radical samples (0.5 g) from the same treatment were taken and immediately frozen in liquid nitrogen for metabolomic analysis. For each treatment, four biological replications were created. To analyze the metabolites in tomato seeding radicals, a non-targeted metabolomic technique was performed using an Agilent 1290 infinity LC ultra-performance liquid chromatography (UHPLC) system (Agilent Technologies, Santa Clara, CA, USA) coupled with AB Sciex TripleTOF 6600 quadrupole time-of-flight detector (AB Sciex, Framingham, MA, USA). The sample preparation, UHPLC-MS analysis, metabolites identification, and quantification were carried out by Personal Biotechnology Co., Ltd. (Shanghai, China) with their standard procedures [29]. For the metabolites, multivariate analysis in the three treatments, the Pareto-scaled principal component analysis (PCA), and orthogonal partial least-squares discriminant analysis (OPLS-DA) were utilized. Differential accumulation metabolites (DAMs) were identified with variable significance in the projection (VIP) value of OPLS-DA ≥ 1, *p*-value < 0.05, and log 2 (Fold Chang) > 1. Finally, the DAMs were annotated to the corresponding metabolic pathways by utilizing the Kyoto Encyclopedia of Genes and Genomes (KEGG) pathway analysis on MetaboAnalyst (http://www.metaboanalyst.ca/, accessed on 5 December 2022).

### 2.7. Transcriptome Analysis

Seven-day-cultivated tomato seeds from the CK, *p*-CA, and *p*-CA + BDOp treatments were also employed for transcriptome analysis with four biological replications. Total RNA was extracted from 0.2 g radical samples using the Trizol Reagent (Invitrogen Life Technologies Corporation, Carlsbad, CA, USA), and the concentration, purity, and integrity were determined with a NanoDrop spectrophotometer (Thermo Fisher Scientific, Waltham, MA, USA). The TruSeq RNA Sample Preparation Kit was used to create sequencing libraries (Illumina Inc., San Diego, CA, USA). Library fragments were purified using the AMPure XP technology (Beckman Coulter, Brea, CA, USA). Using the Illumina PCR Primer Cocktail, DNA fragments with ligated adaptor molecules on both ends were preferentially selected and specifically enriched in a 15-cycle PCR procedure. Products were purified using the AMPure XP system and quantified by the Agilent high-sensitivity DNA assay on a Bioanalyzer 2100 system (Agilent Technologies, Palo Alto, CA, USA). The sequencing library was then conducted on an Illumina Hiseq platform by Personal Biotechnology Co., Ltd. (Shanghai, China). Based on the alignment findings, the expression levels of each gene were assessed after quality control and data cleaning of the gathered raw data. The samples were then subjected to expression difference, enrichment, and cluster analyses. In order to identify the primary biological activities of the enriched differentially expressed genes (DEGs) (*p* < 0.05), cluster profilers were employed for the KEGG (http://www.kegg.jp, accessed on 19 January 2023) enrichment investigation.

### 2.8. Quantitative Real-Time PCR (qRT-PCR) Analysis

To validate the transcriptome results, 5 representative DEGs were subjected to a quantitative real-time polymerase chain reaction (qRT-PCR). RNA was isolated and reverse-transcribed into cDNA using Plant Total RNA Isolation Kit (Sangon Biotech (Shanghai) Co., Ltd., Shanghai, China), Glyceraldehyde 3-phosphate dehydrogenase (GAPDH) was employed as an internal reference gene, and real-time fluorescence quantitative PCR detection was performed with a CFX96 RealTime PCR Detection System (Bio-Rad). Individual gene-specific primers are listed in Table S2.

### 2.9. Statistical Analysis

One-way analysis of variance (ANOVA) was performed using SPSS statistical software (version 21.0, IBM Corp., Armonk, NY, USA). Differences between samples were examined using the least significant difference (LSD) test at the 0.05 level (*p* < 0.05). The data were presented as the means ± standard deviation (SD). The graphics were created using Origin9.0 (OriginLab Co. Northampton, MA, USA), R software packages, and Genescloud tools (https://www.genescloud.cn/home accessed from 15 June 2022 to 23 February 2023). Redundancy analysis (RDA) was performed by using CANOCO 5.0 software (Microcomputer Power, Ithaca, NY, USA).

## 3. Results

### 3.1. Effect of BDO Composite Particles on Tomato Seed Germination

**Figure 1:**
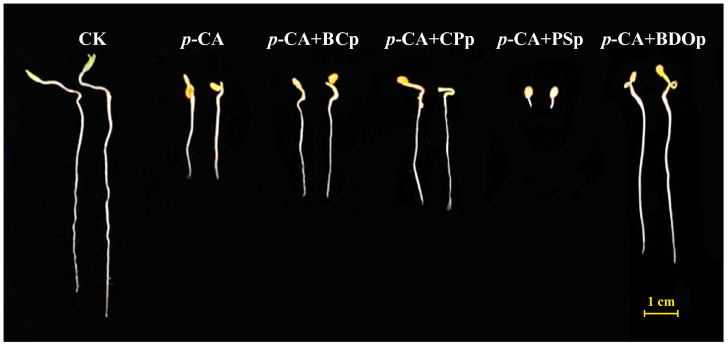
The phenotypes of tomato seeds at 7 days of germination under different treatments. Abbreviations: CK, control check with ddH_2_O; *p*-CA, stress treatment with 10 mL 400 mg/L *p*-coumaric acid; *p*-CA + BC_P_, 10 mL 400 mg/L *p*-coumaric acid with 0.0250 g biochar particles; *p*-CA + CP_P_, 10 mL 400 mg/L *p*-coumaric acid with 0.0027 g calcium peroxide particles; *p*-CA + PS_P_, 10 mL 400 mg/L *p*-coumaric acid with 0.0098 g potassium persulfate particles; *p*-CA + BOD_P_, 10 mL 400 mg/L *p*-coumaric acid with 0.0375 g of biochar-dual oxidant particles.

In order to explore the possibility of eliminating *p*-CA stress, we investigated the effect of different particles on tomato seed germination. Four different types of particles were applied, i.e., biochar particles (BCp), calcium peroxide particles (CPp), potassium persulfate particles (PSp), and biochar-dual oxidant particles (BDOp). Compared to the control (CK), the application of 400 mg/L *p*-CA significantly decreased the radical length (RL) of tomato seedlings by an average of 58.2% at 7 days post-germination (Figure 1 and Table 1). Furthermore, the addition of exogenous *p*-CA reduced the radical length (RL), radical surface area (RSA), and germination index (GI) of seeds by 2.6%, 38.0%, and 62.4% in comparison to CK, respectively. The negative synthetical allelopathic effect index (SE) value for *p*-CA treatment indicated that 400 mg/L *p*-CA inhibited the germination of tomato seeds.

Biochar, calcium peroxide, and potassium persulfate are the principal constituents of BDO composite particles. The RL, RSA, and GI increased by 19.8%, 10.3%, and 30.3%, respectively, by priming with BC particles under *p*-CA stress compared to that of *p*-CA stress treatment (Table 1). Similar results were also obtained with the addition of CP particles, while PS particles had a significant inhibitory impact on tomato seed germination, which was even stronger than the effect of phenolic acid stress alone. Noteworthy, all the measured germination indices of adding BDO composite particles were improved compared to that of adding the other three components (biochar, calcium peroxide, and potassium persulfate) individually. Compared to *p*-CA treatment, BDO particle application enhanced the RL, RSA, and GI by approximately 95%, 53%, and 115%, respectively, and the SE value is also close to zero. In addition, we also investigated the effects of two-component composite particles, i.e., BC-CP composite particles (BC-CPp), BC-PS composite particles (BC-PSp), and CP-PS composite particles (CP-PSp), on the germination of tomato seeds under *p*-CA stress. None of them performed better on seed germination than the BDO composite particles (Table S3). These results indicated that the combination of biochar, calcium peroxide, and potassium persulfate had a synergistic improvement effect on tomato seed germination under *p*-CA stress.

### 3.2. Effect of BDO Composite Particles on the Removal of p-CA

**Figure 2:**
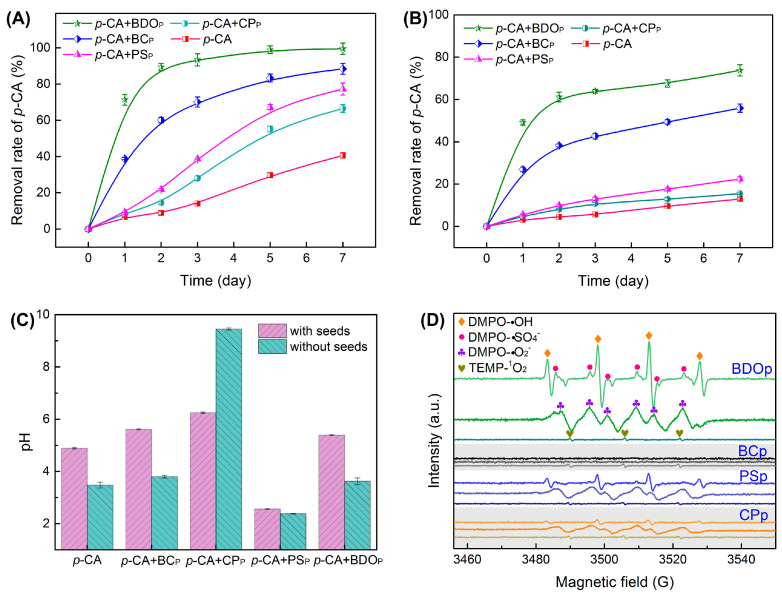
Evolution of the *p*-CA removal rate was determined under different treatments with tomato seeds (**A**) and without seeds (**B**). The pH value of the solution was measured under different treatments on the 7th day after culture (**C**). Results presented as the mean ± SD (*n* = 4). (**D**) Electron paramagnetic resonance (EPR) spectra of reactive oxidizers with DMPO and TEMP after 10.0 min of reaction (Reaction conditions: 10 mL 400 mg/L *p*-CA; amount for BDO, BC, PS, and CP particles were 0.0375, 0.0250, 0.0098, and 0.0027 g, respectively; reaction temperature was 25 °C). Treatment abbreviations: CK, control check with ddH_2_O; *p*-CA, *p*-coumaric acid stress treatment; *p*-CA + BC_P_, *p*-coumaric acid with biochar particles; *p*-CA + CP_P_, *p*-coumaric acid with calcium peroxide particles; *p*-CA + PS_P_, *p*-coumaric acid with potassium persulfate particles; *p*-CA + BOD_P_, *p*-coumaric acid with biochar-dual oxidant particles.

To assess the effectiveness of BDO composite particles in removing phenolic acid, the degradation efficiency of *p*-CA during tomato seed germination was assessed at 1, 2, 3, 5, and 7 days after culture. The removal rates of *p*-CA in the four treatments with the addition of exogenous particles were higher than that of the *p*-CA treatment alone (Figure 2A). The addition of BDO particles achieved the highest *p*-CA removal rate, reaching 99.5% on the 7th day, followed by BC particles (88.4%), PS particles (77.2%), and CP particles (66.5%).

Furthermore, the *p*-CA removal rates in each treatment without tomato seeds were examined. There was a decrease in the removal rate of 25.7–54.8% when compared to each treatment with tomato seeds (Figure 2B), suggesting that *p*-CA can be partially metabolized by the seeds during germination. The addition of BDO particles also presented the highest *p*-CA removal rate (73.8% on the 7th day), followed by BC particles (54.9%), PS particles (22.4%), and CP particles (15.5%).

The pH of the solution was also measured on the 7th day after culture (Figure 2C). The pH of the solution dropped to 2.56 and 2.38 after the addition of PS particles with and without tomato seeds. While the CP particle addition increased the pH to 6.25 and 9.45 in the presence and absence of tomato seeds. In the BDO composite particle addition system, the pH was close to neutral. As a result, the combination of biochar, calcium peroxide, and potassium persulfate in BDO particles successfully prevented the pH from becoming overly alkaline or acidic when compared to adding PS or CP alone, which was also in accord with a greater phenolic acid elimination rate.

The EPR experiments were performed to further confirm the presence and contributions of reactive oxygen species generated during the degradation of *p*-CA by various particles (without the presence of tomato seeds). In the BDO particle system, we detected significant splitting signals with relative intensity ratios of 1:1:1:1 and 1:2:2:1 quartet signals, as well as a six-line signal, which were assigned to the distinctive spectra of DMPO-O_2_^•−^, DMPO-HO^•^ and DMPO-SO_4_^•−^ (Figure 2D). Meanwhile, a modest 1:1:1 triplet signal for TEMP-^1^O_2_ was also observed in the BDO particle system. While no discernible signal for O_2_^•−^, HO^•^, and SO_4_^•−^ was observed with the BC particle addition, indicating that *p*-CA elimination in the BC particle system was mostly due to adsorption rather than oxidation. Characteristic signals of O_2_^•−^, HO^•^, SO_4_^•−^, and ^1^O_2_ were also identified in the systems with the addition of PS and CP particles; however, the peak intensity was remarkably lower than that in the BDO particle system (Figure 2D). These results demonstrated that the combination of biochar, calcium peroxide, and potassium persulfate was favorable for peroxide activation, strengthening the production of strong oxidative free radicals, which was beneficial for the removal of *p*-CA.

### 3.3. Effect of BDO Composite Particles on Antioxidant Enzyme Activity and Lipid Peroxidation

**Figure 3:**
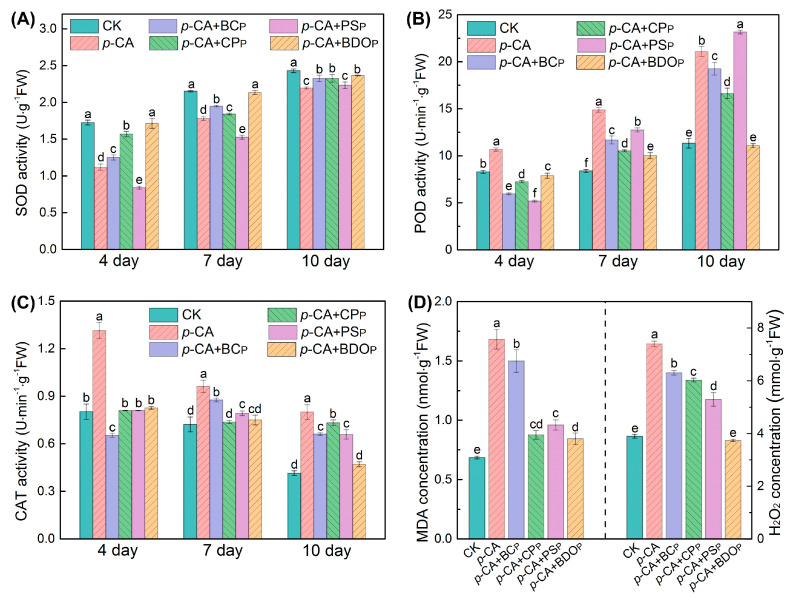
Effects of various treatments on the enzymatic activities of superoxide dismutase (SOD) (**A**), peroxidase (POD) (**B**), and catalase (CAT) (**C**) in tomato seed radicals. The contents of malondialdehyde (MDA) and H_2_O_2_ were measured in tomato seed radicals on the 7th day of culture (**D**). Treatment abbreviations: CK, control check with ddH_2_O; *p*-CA, stress treatment with 10 mL 400 mg/L *p*-coumaric acid; *p*-CA + BC_P_, *p*-coumaric acid with biochar particles; *p*-CA + CP_P_, *p*-coumaric acid with calcium peroxide particles; *p*-CA + PS_P_, *p*-coumaric acid with potassium persulfate particles; *p*-CA + BOD_P_, *p*-coumaric acid with biochar-dual oxidant particles. Bars represent mean ± SD (*n* = 4). Different letters indicate significant statistical differences between treatments (One-way ANOVA, LSD test, *p* < 0.05).

To better understand the effect of BDO composite particles in reducing phenolic acid oxidative stress on tomato seeds, the typical antioxidant enzymes, including SOD, POD, and CAT, were measured. The activities of SOD and POD in the tomato seed radicals of each treatment (Figure 3A,B) increased with the culture time, but the activity of the CAT enzyme gradually reduced (Figure 3C). Treatment with *p*-CA significantly reduced SOD activity in tomato roots, while dramatically increasing POD and CAT activity (*p* < 0.05). Among all the different particles tested here, the BDO composite particle was the best candidate for eliminating the effect of *p*-CA on SOD, POD, and CAT enzymatic activity. For instance, on the 7th day of culture, there was no significant difference in SOD and CAT activities in tomato radicals for the BDO particles addition group compared to the control, and ca. 32.7% decrease in POD activity was observed compared to *p*-CA stress treatment alone.

To further investigate the extent of oxidative stress and membrane lipid peroxidation damage, the contents of MDA and H_2_O_2_ in tomato seed radicals were measured on the 7th day of culture (Figure 3D). As compared to the control, *p*-CA stress markedly increased the amounts of MDA and H_2_O_2_ in tomato radicals by 2.5 and 1.9 times, respectively. The introduction of four types of exogenous particles decreased the rise in MDA content and the accumulation of H_2_O_2_ to some extent under phenolic acid stress. When compared to the *p*-CA treatment alone, the BDO composite particles addition group achieved the greatest reductions in MDA and H_2_O_2_ by 49.7% and 49.5%, respectively. Furthermore, the content of MDA in the group treated with BDO granules was only 18.8% higher than that in the control group, and the level of H_2_O_2_ was not significantly different. Hence, the addition of BDO compound granules can effectively alleviate the membrane peroxidation in tomato seedlings caused by exogenous *p*-CA.

Furthermore, in order to reveal the correlation between the H_2_O_2_ and MDA quantities, SOD, CAT, and POD enzyme activities in the tomato seed radical, a Spearman correlation analysis was performed based on the results of CK and five treatment groups on the 7th day of culture (Figure S1). The concentration of H_2_O_2_ in tomato seed radicles was significantly positively correlated with MDA level, CAT, and POD activity, but negatively correlated with SOD activity. MDA content was positively correlated with CAT and POD activity (*p* < 0.01).

### 3.4. Metabolomic Analysis of Tomato Radicals in Response to BDO Composite Particles during Seed Germination

To identify the metabolites in tomato radicles for the ddH_2_O control group (CK), *p*-CA stress group (labeled as *p*-CA), and BDO particles addition group (abbreviated as BDOp), UHPLC-MS analysis was used in this work. A total of 1052 metabolites were discovered, including 720 and 332 compounds identified in the positive and negative ion modes, respectively. All identified metabolites were classified into 11 categories (Figure S2A), among them, lipids and lipid-like molecules were the most abundant metabolites, accounting for approximately 29.8%, followed by phenylpropanoids and polyketides (13.0%), organoheterocyclic compounds (9.6%), benzenoids (8.8%), organic acids and derivatives (8.7%), and organic oxygen compounds (8.4%).

**Figure 4:**
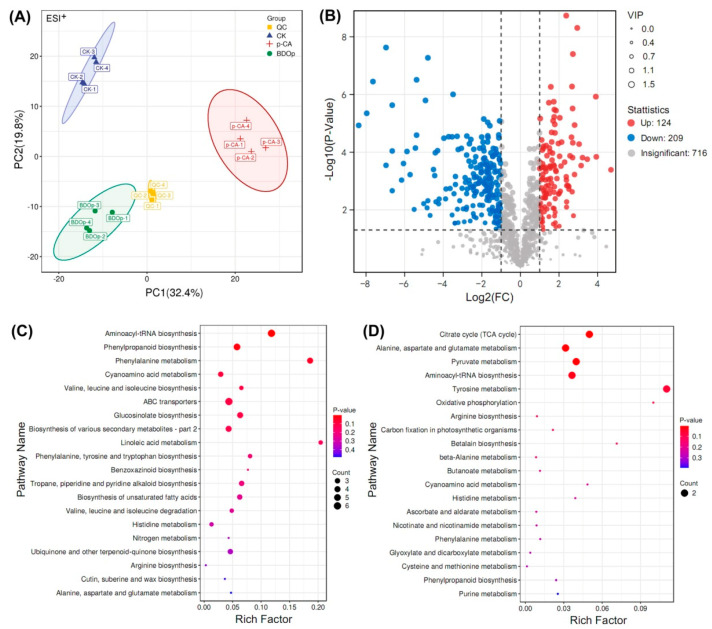
Principal component analysis of all detected metabolites in different treatment groups under positive (ESI^+^) ion mode (**A**). (QC: quality control; CK: ddH_2_O control group; *p*-CA: treatment group with 10 mL 400 mg/L *p*-coumaric acid; BDOp: *p*-coumaric acid with biochar-dual oxidant particles. Each treatment was subjected to four biological replicates). Volcano map of metabolites in CK vs. *p*-CA group (**B**). The bubble diagram for the top 20 significantly enriched KEGG pathways of the 224 (165 up- and 59 down-regulated) differentially accumulated metabolites (DAMs) in response to the *p*-CA stress (**C**) and 44 (19 up- and 25 down-regulated) DAMs specifically regulated by the BDO composite particles (**D**).

PCA and OPLS-DA multivariate analysis were used to analyze the repeatability of the metabolomes in the examined treatments for both positive ion mode and negative ion mode (Figure 4A and Figure S2B–F). In PCA score plots (Figure 4A), the samples from the same group were clustered together. In addition, samples from the *p*-CA group were separated from the CK or BDO particle addition groups. These findings indicated that the groups had high repeatability, with substantial variations in metabolites detected in the *p*-CA stress group compared to the CK and BDOp groups. Furthermore, an OPLS-DA analysis and cross-validation test were performed (Figure S2G,H), The obtained quality parameters (R^2^Y) of OPLS-DA models were close to 1.0, indicating that the fitting results were accurate and the models were reliable.

Based on the results of the OPLS-DA model analysis, the differentially accumulated metabolites (DAMs) with variable important in projection (VIP) ≥ 1, *p*-value < 0.05, and log 2 (Fold Chang) > 1 were selected. The numbers of up-regulated and down-regulated DAMs in the CK vs. *p*-CA group, *p*-CA vs. BDO particle addition group, and CK vs. BDO particle addition group were displayed in the volcano plots (Figure 4B and Figure S3). Through comparison, 165 common DAMs were up-regulated under *p*-CA stress but down-regulated by the application of BDO particles. In contrast, 59 common DAMs were down-regulated under *p*-CA stress but up-regulated following the addition of BDO particles. A total of 19 metabolites were both up-regulated in the CK vs. BDO particle addition group and the *p*-CA vs. BDO particle addition group, but did not overlap with the DAMs down-regulated in the CK vs. *p*-CA group. On the contrary, 25 DAMs were particularly down-regulated after the addition of BDO particles. The majority of the metabolites were phenylpropanoids and polyketides, lipids and lipid-like molecules, organic acids and derivatives, organic oxygen compounds, and others (Figure S4).

To explore the biological pathways induced by *p*-CA stress and BDO particles in tomato seed radicles, the identified DAMs were subsequently assigned to the Kyoto Encyclopedia of Genes and Genomes (KEGG) pathway database. The KEGG analysis indicated that the 165 up-regulated and 59 down-regulated DAMs in response to the *p*-CA stress were significantly enriched (*p* < 0.05) in 12 pathways, including the amino acid metabolism pathways (e.g., phenylalanine, tyrosine, and tryptophan biosynthesis), unsaturated fatty acid metabolism pathway (e.g., unsaturated fatty acids biosynthesis and linoleic acid metabolism), and the biosynthesis of other secondary metabolites (e.g., phenolopanoid, alkaloid and glucosinolate biosynthesis) (Figure 4C). The 44 metabolites specifically regulated by the BDO composite particles (19 up-regulated and 25 down-regulated) were enriched in the citrate cycle (TCA cycle), 3 metabolic pathways (alanine, aspartate and glutamate metabolism, pyruvate metabolism, tyrosine metabolism), aminoacyl-tRNA biosynthesis, and oxidative phosphorylation (Figure 4D).

### 3.5. Transcriptome Analysis of Tomato Radicals in Response to BDO Composite Particles during Seed Germination

**Figure 5:**
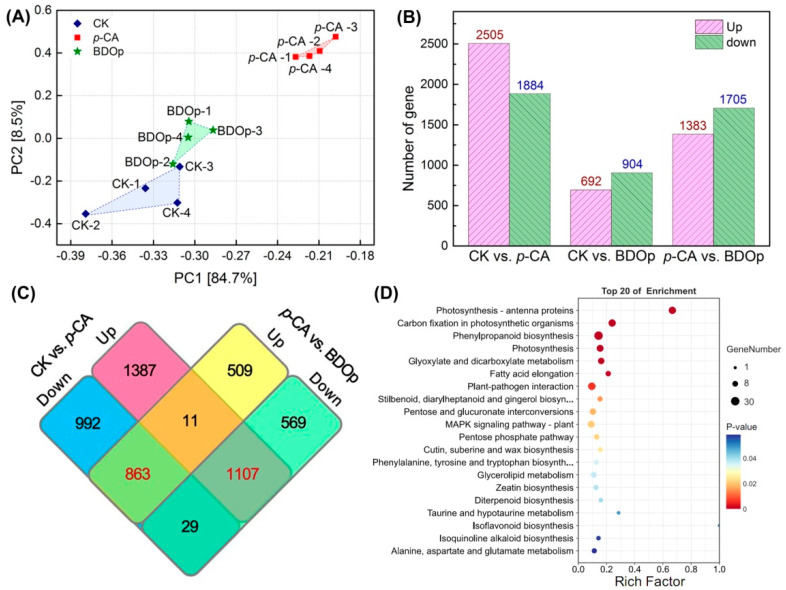
Principal component analysis (PCA) (**A**) of FPKM profiles in three treatment groups; The statistics of the up- and down-regulated DEGs in pairwise comparison of treatments (**B**). Venn diagram of the number of up-regulated and down-regulated DEGs in CK vs. *p*-CA and *p*-CA vs. BDOp pairwise comparison (**C**). The bubble diagrams for the top 20 KEGG pathways for 1107 up-regulated differentially expressed genes (DEGs) in CK vs. *p*-CA group (**D**). (CK: ddH_2_O control group; *p*-CA: treatment group with 10 mL 400 mg/L *p*-coumaric acid; BDOp: *p*-coumaric acid with biochar-dual oxidant particles. Each treatment was subjected to four biological replicates).

The transcriptomes of seeds radicles treated with ddH_2_O, *p*-CA, and BDOp were investigated to determine the internal regulation mechanism of adding BDO compound particles to tomato seed germination under phenolic acid stress. More than 40 million raw reads were obtained from each sample. After quality filtration, the Q30 scores of the clean reads were more than 92.9%, with GC content higher than 42%, and an error rate of less than 0.03%. In addition, the principal component analysis (PCA) (Figure 5A) revealed that principal components 1 (PC1) and PC2 explained 84.7% and 8.5% of the variance, respectively. Samples from three different treatments were clearly separated, and the expression of gene clusters in the CK group was close to the BDOp treatment group, whereas it diverged significantly from the *p*-CA stress group. Similar findings were also observed in the heatmap of gene expression clusters (Figure S5A). In order to confirm the RNA-seq data, we further employed qRT-PCR to evaluate the expression of five highly expressed DEGs. The obtained R^2^ of the log_2_-fold change was 0.9174. These results suggested that the accuracy and quality of the RNA sequencing data were reliable for further analysis.

The differentially expressed genes (DEGs) between the different treatment groups were identified by the DESeq R package with log 2 (Fold Change) > 1 and *p*-value < 0.05. A total of 4389 DEGs were identified comparing the control group (CK) to the *p*-CA stress group, including 2505 up-regulated and 1884 down-regulated DEGs (Figure 5B). Compared to CK, only 1596 DEGs were screened for the BDO particle addition group, of which 692 were up-regulated and 904 were down-regulated. The difference in the number of DGEs between the two control groups (CK vs. *p*-CA and CK vs. BDOp) indicated that phenolic acid stress significantly altered the gene expression in tomato radicles, whereas the addition of BDO compound particles to *p*-CA could effectively regulate the gene expression, simultaneously reducing the number of up-regulated and down-regulated DGEs, and restoring the gene expression to that of the control group.

As illustrated in the Venn diagram (Figure 5C), 1107 commonly expressed DEGs were up-regulated in the CK vs. *p*-CA group but down-regulated in *p*-CA vs. BDOp group, while 863 shared DEGs were down-regulated in the CK vs. *p*-CA group but up-regulated in the *p*-CA vs. BDOp group. Furthermore, we discovered a total of 159 and 259 co-expression DEGs were specifically up-regulated and down-regulated due to the application of BDO composite particles, which were unaffected by *p*-CA.

Next, KEGG pathway enrichment analysis was conducted for the detected DEGs. Notably, 1107 DEGs up-regulated under phenolic acid stress were enriched in 9 pathways (Figure 5D), including energy metabolism pathways (e.g., photosynthesis -antenna proteins, carbon fixation in photosynthetic organisms, photosynthesis), biosynthesis of secondary metabolites (e.g., phylproponoid biosynthesis, stilbenoid, diarylheptanoid, and ginger biosynthesis), carbohydrate metabolism (glyoxylate and dicarboxylate metabolism, pentose and glucuronate interconversions), and lipid metabolism (fatty acid elongation). Notably, 863 down-regulated DEGs under phenolic acid stress (Figure S5B) were significantly enriched in environmental information processing, environmental adaptation, and metabolism pathway. The top 10 enriched KEGG pathways for the 159 and 259 DEGs specifically regulated by BDO composite particles were shown in Figure S5C,D. According to the KEGG enrichment results, phenolic acid stress generally led to the upregulation of genes related to photosynthesis, carbohydrate metabolism, and fatty acid metabolism pathways, while down-regulated genes involved in plant hormone synthesis and amino acid metabolism pathways. The addition of BDO composite particles to *p*-CA could effectively regulate the expression of genes related to the synthesis and metabolism of antioxidant substances, and up-regulate the expression of some genes related to plant hormone synthesis.

### 3.6. Integrative Analyses of Antioxidant Enzyme Activity, Metabolomics, and Transcriptomics

**Figure 6:**
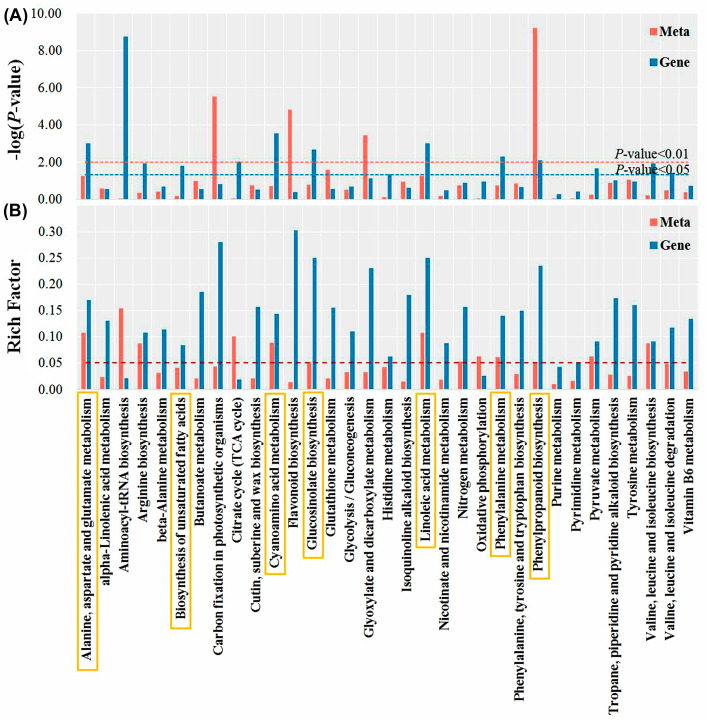
The −log*P*-value (**A**) and rich factor (**B**) correspond to the KEGG pathway enriched by differentially expressed genes (DEGs) and differentially accumulated metabolites (DAMs). The yellow rectangle depicts seven co-enriched metabolic pathways of concern.

Integrated metabolomics and transcriptomics analyses were performed. Notably, 33 KEGG pathways were shared between transcripts and metabolites, among which the phenylpropanoid biosynthesis pathway was significantly enriched (*p*-value < 0.01) (Figure 6). In addition, there were five common enrichment metabolic pathways with rich factors of both transcriptomics and metabolomics greater than 0.05, and a *p*-value of transcriptomics or metabolomics less than 0.01, they were alanine, aspartate and glutamate metabolism, cyanoamino acid metabolism, glucosinolate biosynthesis, linoleic acid metabolism, and phenylalanine metabolism. Considering the unique significance of unsaturated fatty acids in plant stress tolerance, the biosynthesis of the unsaturated fatty acids pathway was also a concern.

**Figure 7:**
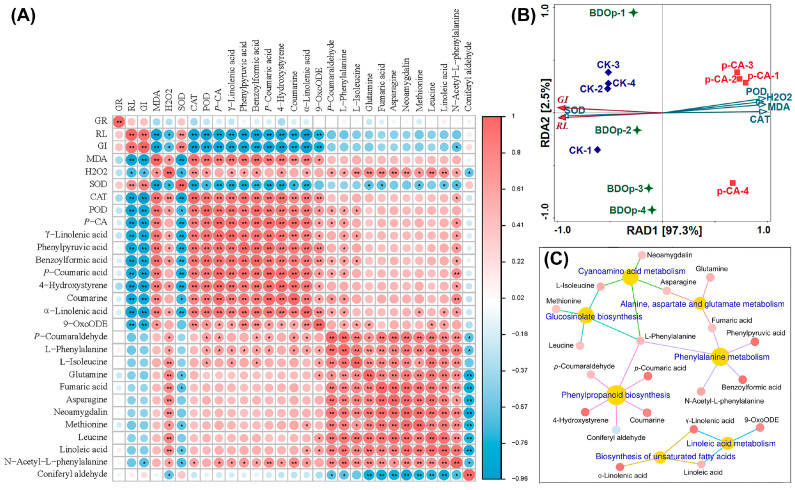
Heatmap analysis between germination indices, antioxidant indices, and 20 differentially accumulated metabolites (DAMs) (**A**). Redundancy analysis of germination indexes and antioxidant index (**B**). Metabolite network for the 20 DAMs in KEGG enriched pathways (**C**). Abbreviations: RL, radical length; GI, germination index; SOD, superoxide dismutase; POD, peroxidase; CAT, catalase; MDA, malondialdehyde; CK, ddH_2_O control group; *p*-CA, treatment group with 10 mL 400 mg/L *p*-coumaric acid; BDOp, *p*-coumaric acid with biochar-dual oxidant particles. * *p* < 0.05 and ** *p* < 0.01.

The above selected 7 pathways involve a total of 20 metabolites. We further analyzed the relationship between seed germination indices, antioxidant enzyme activity, and the 20 key metabolites in further detail. According to the heatmap (Figure 7A), GI and RL were positively correlated (*p* < 0.01) with SOD but negatively correlated with MDA, CAT, and POD. In general, they were also negatively correlated with 19 key metabolites. This demonstrated that phenolic acid stress led to the accumulation of the majority of key metabolites in tomato seeds, as well as an increase in CAT and POD, but a decrease in SOD activity, and the accumulation of MDA in the radicles, which restricted seed growth. The addition of BDO composite particles had the opposite effects.

Redundancy analysis (RDA) was utilized to study the correlation between seed germination indices (RL, GI) and antioxidant indices (SOD, CAT, BOD, MDA, and H_2_O_2_ content in the radicle). The total interpretation degree of the first and second ranking axes in the RDA analysis achieved 99.82% (Figure 7B). The sample points of the CK and BDO composite particle treatment groups, and phenolic acid stress treatment groups were distributed on both sides of the first axis of RDA, revealing that the application of BDO particles could effectively alleviate the phenolic acid stress effect, bringing the sample points closer to CK. The results of RDA also showed that CAT explained 96.6% (*p* = 0.002) of the variance of RL and GI in seed germination, indicating that CAT enzyme activity was the most important physiological factor influencing seed germination. On the other hand, according to the heatmap results (Figure 7A), CAT had a substantial positive connection with nine key DAMs, including 4-hydroxystyrene, phenylpyruvic acid, *p*-coumaric acid, benzoylformic acid, γ-Linolenic acid, coumarine, α-linolenic acid, 9-OxoODE (*p* < 0.01), and N-acetyl-L-phenylalanine (*p* < 0.05). The relative contents of these nine key DAMs were considerably higher in the *p*-CA treatments than in the CK ones. The fold change values were 301.3, 31.9, 29.0, 21.3, 14.4, 9.6, 3.8, 2.4, and 3.0, respectively. While the fold change values decreased to between 0.09 and 0.47 for the BDO particles addition group compared to *p*-CA stress group. It indicated that the relative content of these key DAMs increased dramatically under phenolic acid stress and decreased to a certain extent with the application of BDO composite particles. The association network (Figure 7C) between metabolic pathways and the 20 major DAMs showed that the 9 key DAMs strongly associated with CAT were mainly related to 4 metabolic pathways: phenylpropanoid biosynthesis, phenylalanine metabolism, biosynthesis of unsaturated fatty acids, linoleic acid metabolism, which involving 62 DEGs and 14 DAMs in all.

**Figure 8:**
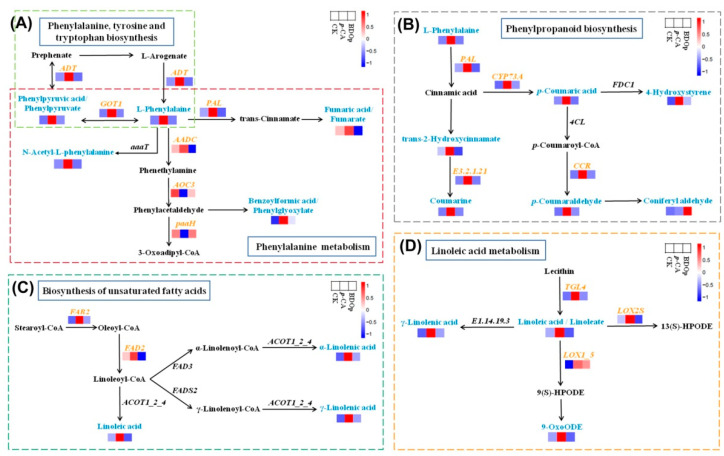
Visual analysis of the network of phenylpropanoid biosynthesis (**A**), phenylalanine metabolism (**B**), biosynthesis of unsaturated fatty acids (**C**), linoleic acid metabolism (**D**) for the CK, *p*-CA stress treatment, and BDOp treatments. (CK, ddH_2_O control group; *p*-CA, treatment group with 10 mL 400 mg/L *p*-coumaric acid; BDOp, *p*-coumaric acid with biochar-dual oxidant particles.)

Among the above 4 key metabolic pathways, phenylpropanoid biosynthesis and phenylalanine metabolism pathways enriched the greatest number of key DEGs and DAMs, i.e., 55 DEGs and 9 DAMs in total. L-phenylalanine and phenylpyruvic acid were significantly up-regulated in the *p*-CA stress treatment in line with up-regulating *ADT* and *GOT1* in the phenylalanine, tyrosine, and tryptophan biosynthesis pathway (Figure 8A), which is the upstream pathway of phenylpropanoid biosynthesis and phenylalanine metabolism. For the phenylalanine metabolism, the levels of N-acetyl-L-phenylalanine, fumaric acid, and benzoylformic acid were up-regulated together with the upregulation of *PAL* and *AADC* in the *p*-CA stress treatment. For the phenylpropanoid biosynthesis pathway (Figure 8B), the levels of *p*-coumaric acid, 4-hydroxystyrene, trans-2-hydroxycinnamate, coumarine, and *p*-coumaraldehyde were also up-regulated in line with up-regulating the expression of *PAL*, *CYP73A*, *E3.2.1.21*, *CCR* under the *p*-CA stress, but exhibited lowered levels in the BDO particles addition treatment compared with those in the *p*-CA treatment.

In addition, phenolic acid stress specifically up-regulated the genes encoding *FAB2* and *FAD2*, promoting the synthesis of linoleic acid, α-linolenic acid, and γ-linolenic acid (Figure 8C). The expression levels of *TGL4*, *LOX2S*, and *LOX1_5* were up-regulated, which accelerated the linoleic acid metabolism to produce γ-Linolenic acid and 9-OxoODE (Figure 8D).

## 4. Discussion

### 4.1. BDO Composite Particles Possess the Ability to Eliminate Phenolic Acids While Stimulating Seed Germination

Seed germination is the critical initial stage of the plant life cycle, affecting subsequent plant development and crop yield [30]. Allelochemicals accumulated in soil by plants inhibit seed germination and seedling development. The oxidative stress caused by phenolic acids as typical allelochemicals has been extensively studied over the past three decades, both in laboratory bioassays and field research [10,31]. The root is the first mediation organ exposed to phenolic acid stress, and its physiological activity may be extremely sensitive during the interaction process. Consequently, the process of seed germination is particularly essential to the study of abiotic oxidative stress. In the present study, *p*-coumaric acid (*p*-CA) stress significantly reduced tomato seed germination rate, radicle length, and germination indices (Figure 1 and Table 1). The inhibitory effect increased as the concentration of *p*-CA increased (Table S1). Numerous investigations have observed a similar phenomenon, i.e., the phenolic acids impeding germination and the growth of bean seed radicles and shoots [32].

Compared to the *p*-CA stress treatment, the addition of BDO composite particles resulted in substantial increases in tomato seed germination and seeding growth in this study (Figure 1 and Table 1). In recent years, biochar has been utilized to eliminate allelochemicals from water and soil. For instance, Karunanayake et al. demonstrated that biochar generated by fast pyrolysis of Douglas fir could rapidly absorb benzoic acid and phthalic acid from wastewater, with much faster adsorption kinetics than pinewood biochar and industrial activated carbon [33]. Gámiz et al. used sorption experiments and soil column leaching studies to determine the effect of biochar addition on the residue amount of coumarine in soil. The results revealed that the total amount of extracted coumarine within the topsoil increased from 1% to between 17% and 22% after biochar amendment [34].

In our investigation, BDO particles showed higher phenolic acid removal performance than biochar particles (Figure 2A,B). EPR studies revealed observable O_2_^•−^, HO^•^, SO_4_^•−^ radicals in the BDO particles system. However, free radicals were not obviously detected in the BC particle system (Figure 2D). Hence, it suggested that the removal of phenolic acid in the BDO composite particle system was attributed to the combined effects of biochar adsorption and free radical oxidation. The results of this study revealed that the removal rate of *p*-CA in the calcium peroxide (CP) and potassium persulfate (PS) particle systems was significantly lower than that of the BDO composite particles system, with lower intensity of free radicals detected by EPR. For peroxides, PS can be triggered by the H_2_O_2_ and the heat generated from the reaction of calcium peroxide with water (Equations (5)–(9)) [35,36]. Simultaneously, acids are released from the decomposition of PS, preventing the inefficient conversion of CaO_2_ to O_2_ in an alkaline environment caused by Ca(OH)_2_. Biochar has been found to have certain activation effects on persulfate based on its unique electron transport capabilities and surface chemical characteristics (Equations (10)–(12)) [21]. In addition, partially formed SO_4_^•−^ can react with water to create HO^•^ (Equation (13)) [37]. It is evident that the combination of BC, CP, and PS may establish a mutual autocatalytic effect between components, increase peroxide utilization efficiency and catalytic activity, as a consequence, achieve a greater phenolic acid elimination rate than a system with a single component. Simultaneously, the composite system successfully avoids severe alkali or acid induced by the application of CP and PS alone and maintains a moderate pH environment, which is beneficial for the germination of tomato seeds.

CaO_2_ + 2H_2_O → Ca(OH)_2_ + H_2_O_2_ (5)

H_2_O_2_ → H_2_O + O_2_ + Heat (6)

S_2_O_8_^2−^ + Heat → 2SO_4_^•−^ (7)

S_2_O_8_^2−^ + H_2_O_2_ → SO_4_^•−^+ HO_2_^•^ + HSO_4_^−^ (8)

HO_2_^•^ → O_2_^•−^ + H^+^ (9)

S_2_O_8_^2−^ + ACsurf-e^−^→ SO_4_^•−^ + SO_4_^2−^ (10)

BCsurf-OH + S_2_O_8_^2−^ → BCsurf-O^•^ + SO_4_^•−^ + HSO_4_^−^ (11)

BCsurf-OOH + S_2_O_8_^2−^ → BCsurf-OO^•^ + SO_4_^•−^ + HSO_4_^−^ (12)

SO_4_^•−^ + H_2_O → ^•^OH + HSO_4_^−^ (13)

### 4.2. Antioxidant Enzyme Activity in Tomato Seeds Rescued by BDO Composite Particles under Phenolic Acid Stress

Previous studies have demonstrated that phenolic acid stress can cause over-accumulation of reactive oxygen species (ROS) in plants [38]. Excessive formation of ROS, such as hydrogen peroxide (H_2_O_2_), induces membrane lipid peroxidation and MDA production, leading to membrane structural damage and even programmed cell death [39]. In our study, a significant increase was found in H_2_O_2_ and MDA content in tomato seed radicals on the 7th day of culture (Figure 3D), indicating that the *p*-CA used in this experiment induced severe oxidative stress and membrane lipid peroxidation in tomato seeds, thereby inhibiting seed germination and radicle growth.

The antioxidant defense system is critical in the scavenging and detoxification of stress-induced ROS [40]. This defense system contains both enzymatic and nonenzymatic antioxidants, with the former predominantly consisting of superoxide dismutase (SOD), catalase (CAT), and peroxidase (POD) [41,42]. SOD is the first antioxidant enzyme capable of rapidly converting superoxide radicals (O_2_^•−^) generated by seeds under oxidative stress in H_2_O_2_. CAT and POD then further degrade the H_2_O_2_ into H_2_O and O_2_ [43,44]. Our results showed that the activities of CAT and POD in tomato radicles were induced under *p*-CA stress (Figure 3). This demonstrated that the antioxidant defense capacity of tomato seeds was enhanced in order to eliminate excess ROS produced by stress and maintain embryo development. While *p*-CA stress lowering SOD activity may be due to the accumulation of excess H_2_O_2_ in tomato seeds, which would inhibit the conversion of O_2_^•−^ to H_2_O_2_ catalyzed by SOD, resulting in reduced SOD activity. However, as the culture experiment progressed, H_2_O_2_ decomposed gradually and SOD activity increased. The association analysis of enzyme activity with H_2_O_2_ and MDA contents (Figure S1) revealed that CAT had the most significant positive correlation with H_2_O_2_ content. Shi et al. demonstrated that CAT played a more important role than other enzymes in scavenging excess H_2_O_2_ under stress conditions [45]. Moles et al. also discovered that salt stress increased the CAT of Ciettaicale Italian tomato seeds, which was beneficial for the removal of accumulated H_2_O_2_ [46]. These findings are consistent with our study.

With the application of BDO composite particles, the content of H_2_O_2_ and MDA and the CAT activity in the radicles of tomato seeds decreased, while the SOD activity increased significantly in comparison to the *p*-CA treatment (Figure 3). The results showed that the addition of BDO particles could regulate the antioxidant enzyme system of tomato seeds, alleviate oxidative stress caused by phenolic acid and prevent membrane lipid peroxidation. Wang et al. demonstrated that adding 80 g/kg biochar to continuous cropping soil could effectively improve SOD activity in the leaves of *M. hupehensis* Rehd. seedlings by 12% compared to the control, thereby reducing the amount of MDA, H_2_O_2_, and the generation rate of O_2_^•−^ by 50%, 25%, and 71%, respectively. Furthermore, after adding biochar, phenolic acid removal rates in soil ranged from 17% to 42% [17]. Shen et al. proved that carbon nanoparticles could alter the activity of antioxidant enzymes and lower the concentration of MDA in *Oryza sativa* L. cv. rice seeds, thus reducing ferulic acid stress and promoting rice seedling growth [47]. In our study, the regulatory effect of biochar particles and BDO composite particles on antioxidant enzyme activity in tomato seed radicles was found to be opposite to that of *p*-CA stress treatment, and the content of H_2_O_2_ and MDA in the radicle of BDO particles treatment was significantly lower than that of biochar particles treatment. According to the above findings, we demonstrated for the first time that BDO composite particles can more efficiently remove phenolic acids in the culture medium than biochar particles, maintain the balance of antioxidant enzyme activity in the system, alleviate membrane lipid peroxidation, and effectively ensure normal tomato seed germination. Hence, BDO composite particles have promising application prospects in allelochemical reduction and continuous cropping obstacle soil improvement.

### 4.3. BDO Composite Particles Regulate the Metabolism and Transcription of Tomato Seeds under Phenolic Acid Stress

In this study, the combined analysis of metabolism and transcriptome showed that *p*-CA stress and BDO composite particle mitigation significantly regulated phenylpropanoid biosynthesis, phenylalanine metabolism, linoleic acid metabolism, and biosynthesis of the unsaturated fatty acids pathway during tomato seed germination. The phenylpropanoid biosynthesis pathway belongs to the nonenzymatic antioxidant system. Many phenylpropanoids possess potent antioxidative activities against ROS free radicals, hence protecting plant cells from oxidative damage [48]. Under *p*-CA stress, L-phenylalanine was catalyzed to cinnamic acid by up-regulating *PAL* via non-oxidative deamination. In the subsequent step, more cinnamic acid was converted into *p*-coumaric acid through 4-hydroxylation by up-regulating *CYP73A*. The production of monophenol or flavonoids occurs via two critical downstream pathways with an enzyme system consisting of *4CL*, *CCR*, and *FDC1*, resulting in considerable increases in the levels of 4-hydroxystyrene and *p*-coumaraldehyde. In addition, cinnamic acid was rapidly converted to trans-2-hydroxycinnamate under the catalysis of cinnamate 2-hydroxylase, followed by the generation of coumarine through the upregulation of β-glucosidase. Studies have shown that upregulation of *PAL*, *C4H*, and *4CL* in plants can increase plant tolerance to biotic or abiotic stress [49,50]. Among them, overexpression of *PAL* has been proven to reduce the level of ROS in *Arabidopsis* seeds and leaves, increase the activity of POD, CAT, and the content of flavonoids, and reduce the accumulation of MDA [51]. In this study, the relative concentration of *p*-coumaric acid and 4-hydroxystyrene increased dramatically under *p*-CA stress but dropped significantly with BDO particle additions. For the phenylalanine metabolism, the expression level of *ADT* (DEG) was up-regulated to promote the conversion of L-arogenate to phenylalanine in this study (Figure 8), as well as promoted the conversion of prephenate to phenylpyruvic acid. *GOT1* was also up-regulated to accelerate the interconversion between phenylalanine and phenylpyruvic acid. Phenylalanine can serve as an antioxidant and osmotic substance, which helps plants tolerate stress by regulating the osmotic pressure and scavenging free radicals [52]. Additionally, the accumulation of phenylalanine under external stress has the capacity to stimulate glycolysis, activate biosynthetic pathways such as phenylpropanoids, and neutralize destructive ROS through the accumulation of various phenolic compounds [53]. Consequently, we speculate that *p*-CA treatment induces higher biosynthesis and metabolism of phenylalanine and phenylpropanoid, resulting in the accumulation of some specific phenylpropanoids, which ultimately contributes to an increase in antioxidant capacity.

In this study, we found that *p*-CA stress significantly increased the content of γ-Linolenic acid, linolenic acid, linoleic acid, and 9-OxoODE in the roots of tomato seeds, whereas the addition of BDO composite particles greatly lowered the content. These four DAMs are involved in two metabolic pathways, namely the biosynthesis of unsaturated fatty acids and the linoleic acid metabolism pathway. In the biosynthesis of the unsaturated fatty acids pathway, the expression of the *FAB2* gene (DEG) was considerably elevated by *p*-CA stress and dramatically down-regulated by BDOp additions. *FAB2* is a crucial enzyme in the production of linolenic acid and catalyzes the conversion of saturated fatty acids to unsaturated fatty acids [54,55]. The high content of unsaturated fatty acids in the cell membrane is of great significance in maintaining the fluidity of the cell membrane, stabilizing the compartmentalization function of the cell membrane system and the permeability of the membrane. In the linoleic acid metabolism pathway, exogenous BDO particle addition might inhibit genes that are up-regulated in response to stress including *TGL4* and *LOX1_5*. Wang et al. also discovered that under drought stress, five *TGL* genes were up-regulated during tomato seed germination and seedlings growth [56]. *LOX* participates in the oxidation process of unsaturated fatty acids and produces oxygenates via enzymatic or nonenzymatic reactions, which play a crucial role in the regulation of plant growth, development, and stress response [57].

Linoleic acid and its metabolites (γ-linolenic acid, linolenic acid, 9-OxoODE) are the principal components of membrane lipid fatty acids. In addition to basic energy metabolism, they have various functions in plant growth and stress responses [58]. Previous research has revealed that plants exposed to salt, cold, and ion stress synthesize more linolenic acid and its metabolites, and CAT enzyme activity increases dramatically [59,60]. Among them, linolenic acid is oxidized by ROS to MDA under an oxidative stress process [61]. Therefore, linolenic acid is an antioxidant. However, high levels of linolenic acid can also cause oxidative stress in plants. Xu et al. found that a high concentration of linolenic acid increased the amount of ROS in green algae and caused membrane lipid peroxidation [62].

Our results demonstrated that exogenous *p*-CA stress led to overexpression of genes in phenylalanine, phenylpropanoid synthesis, and linoleic acid metabolism pathway in tomato seed radicles, resulting in a substantial accumulation of antioxidant substances to eliminate ROS induced by *p*-CA stress. Exogenous BDO composite particles can effectively remove the *p*-CA from the culture medium, alleviate the stress response of tomato seed at the transcriptional and metabolic levels, and prevent the membrane lipid peroxidation damage under phenolic acid stress.

## 5. Conclusions

The application of biochar-dual oxidant (BDO) composite particles could improve the germination rate and promote radical growth when tomato seeds were exposed to 400 mg/L *p*-coumaric acids (*p*-CA). This promotion effect was mainly due to the fact that the BDO composite particles could produce more O_2_^•−^, HO^•^, SO_4_^•−^ and ^1^O_2_ radicals via the catalytic interaction between the components. By BDO particles with the virtues of biochar adsorption and free radical oxidation, the *p*-CA in the culture medium could be effectively removed, and the pH was maintained at neutral, providing a favorable environment for tomato seed germination. The activity of defensive enzyme SOD was also induced after BDO particle addition, while CAT and POD activity decreased. The MDA and H_2_O_2_ content was also reduced to a relatively normal level in BDO particle treatment under *p*-CA stress. In order to alleviate the oxidative stress from high concentrations of phenolic acid, tomato seeds up-regulated the genes in phenylalanine, phenylpropanoid synthesis, and linoleic acid metabolic pathway, and increased the accumulation of antioxidant and osmotic pressure regulating substances, including phenylpropanoids, linoleic acid, and its metabolites. The expression levels of those genes and the content of metabolites decreased significantly after BDO particle additions.

It showed that BDO particles could effectively alleviate the oxidative stress effect on tomato seeds by removing phenolic acids from the culture medium and restoring the physiological functions of tomato seeds. Overall, our findings suggest that BDO composite particles are potential materials used to protect crops against phenolic acid stress. Since phenolic acid accumulation in the soil is one of the primary causes of continuous cropping obstacles, the high removal efficiency and seed germination promotion effects of BDO composite particles indicate that such materials are very promising to be used for continuous cropping soil amendment.

## Figures and Tables

**Table 1:** Effects of different treatments on tomato seed germination indices.

Treatment	GP (%)	GR (%)	RL (mm)	RSA (cm^2^)	GI (%)	SE
CK	86.5 ± 1.9 a	95.0 ± 2.6 a	61.7 ± 1.9 a	0.468 ± 0.023 a	100.0 ± 0.0 a	0.0 ± 0.0 a
*p*-CA	0.0 ± 0.0 e	92.5 ± 3.4 a	25.8 ± 1.0 e	0.290 ± 0.006 d	37.6 ± 0.6 e	−0.53 ± 0.01 e
*p*-CA + BC_P_	38.0 ± 1.6 d	93.0 ± 2.6 a	30.9 ± 1.2 d	0.320 ± 0.015 c	49.0 ± 1.1 d	−0.38 ± 0.00 d
*p*-CA + CP_P_	62.0 ± 1.6 c	92.0 ± 2.3 a	33.3 ± 2.0 c	0.335 ± 0.021 c	52.2 ± 2.0 c	−0.31 ± 0.00 c
*p*-CA + PS_P_	0.0 ± 0.0 e	29.0 ± 1.2 b	2.2 ± 0.1 f	0.046 ± 0.002 e	1.1 ± 0.0 f	−0.91 ± 0.00 f
*p*-CA + BOD_P_	71.5 ± 3.4 b	94.0 ± 1.6 a	50.3 ± 1.7 b	0.443 ± 0.020 b	80.7 ± 3.9 b	−0.12 ± 0.01 b

Abbreviations: CK, control check with ddH_2_O; *p*-CA, stress treatment with 10 mL 400 mg/L *p*-coumaric acid; *p*-CA + BC_P_, 10 mL 400 mg/L *p*-coumaric acid with 0.0250 g biochar particles; *p*-CA + CP_P_, 10 mL 400 mg/L *p*-coumaric acid with 0.0027 g calcium peroxide particles; *p*-CA + PS_P_, 10 mL 400 mg/L *p*-coumaric acid with 0.0098 g potassium persulfate particles; *p*-CA + BOD_P_, 10 mL 400 mg/L *p*-coumaric acid with 0.0375 g biochar-dual oxidant particles. GP, germination potential; GR, germination rate; RL, radical length; RSA, radical surface area; GI, germination index; SE, synthetical allelopathic effect index. Each data presents the mean ± standard deviation (*n* = 4). Different letters indicate statistically significant differences between treatments (One-way ANOVA, LSD test, *p* < 0.05).

## Data Availability

Data is contained within this article and Supplementary Materials.

## Supplementary Materials

The following supporting information can be downloaded at https://www.mdpi.com/article/10.3390/antiox12040910/s1. Figure S1. Spearman correlation between the content of MDA, H_2_O_2_, and enzymatic activities of SOD, CAT, and POD in tomato seed radicals on the 7th day of culture. Figure S2. Pie chart of metabolite categorization (**A**). PCA score plot of metabolites under negative (ESI^−^) ion mode (**B**). OPLS-DA plots of metabolites in three different treatment groups under positive (ESI^+^) (**C**) and negative (ESI^−^) (**D**) ion mode. OPLS-DA score plot of metabolites in CK and *p*-CA group (**E**) and *p*-CA and BDOp group (**F**). Permutation test of OPLS-DA for CK vs. *p*-CA (**G**) and *p*-CA vs. BDOp (**H**). Figure S3. Volcano map of metabolites in *p*-CA vs. BDOp (**A**) and CK vs. BDOp group (**B**). Figure S4. The sunburst chart for classification of the 165 up-regulated (**A**) and 59 down-regulated (**B**) DAMs in response to the *p*-CA stress, and 19 up-regulated (**C**) and 25 down-regulated (**D**) DAMs specifically regulated by the BDO composite particles. Figure S5. Hierarchical clustering heatmap of FPKM profiles in three treatment groups (**A**). The bubble diagrams for the top 20 KEGG pathways for 863 down-regulated DEGs in response to the *p*-CA stress (**B**), and 159 up-regulated (**C**) and 259 down-regulated (**D**) DEGs specifically regulated by the BDO composite particles. Table S1. Effect of different *p*-coumaric acid concentrations on the germination of tomato seeds. Table S2. Specific primers were used for q-PCR. Table S3. Effects of various treatments on the tomato seed germination indices.
