# Supplementary material for: Biochar-Dual Oxidant Composite Particles Alleviate the Oxidative Stress of Phenolic Acid on Tomato Seed Germination
Source: Antioxidants (Basel). 2023 Apr 11;12(4):910. doi: 10.3390/antiox12040910 (PMC10136075; doi:10.3390/antiox12040910)
Supplement: Supplementary file 1 [file antioxidants-12-00910-s001.zip › antioxidants-2308211-supplementary.pdf]

antioxidants

MDPI

# Supplementary data

# Biochar-dual oxidant composite particles alleviate the oxidative stress of phenolic acid on tomato seed germination

**Yuting Tu [1,3,4,†], Jinchun Shen [2,†], Zhiping Peng [1,3,4], Yanggui Xu [1,3,4], Zhuxian Li [1,3,4], Jianyi Liang [1,3,4], Qiufang Wei [5], Hongbo Zhao [2,*] and Jichuan Huang [1,3,4,*]**

1 Institute of Agricultural Resources and Environment, Guangdong Academy of Agricultural Sciences, Guangzhou 510640, China
2 College of Horticulture, South China Agricultural University, Guangzhou 510642, China
3 Key Laboratory of Plant Nutrition and Fertilizer in South Region, Ministry of Agriculture, Guangzhou 510640, China
4 Guangdong Key Laboratory of Nutrient Cycling and Farmland Conservation, Guangzhou 510640, China
5 College of Natural Resources and Environment, South China Agricultural University, Guangzhou 510642, China
* Correspondence: zhao@scau.edu.cn (H. Zhao); huangjichuan@gdaas.cn (J. Huang)
† These authors contributed equally to this work.

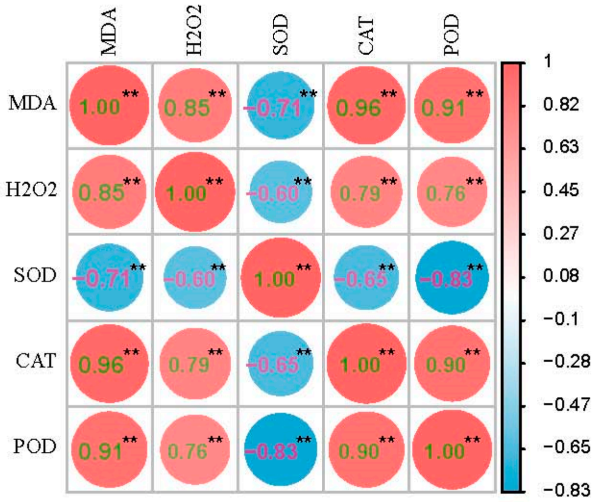


**Figure S1**. Spearman correlation between content of MDA, $H_2O_2$, and enzimatic activities of SOD, CAT and POD in tomato seed radicals on the 7th day of culture. Abbreviations: MDA, malondialdehyde; SOD, superoxide dismutase; CAT, catalase; POD, peroxidase.

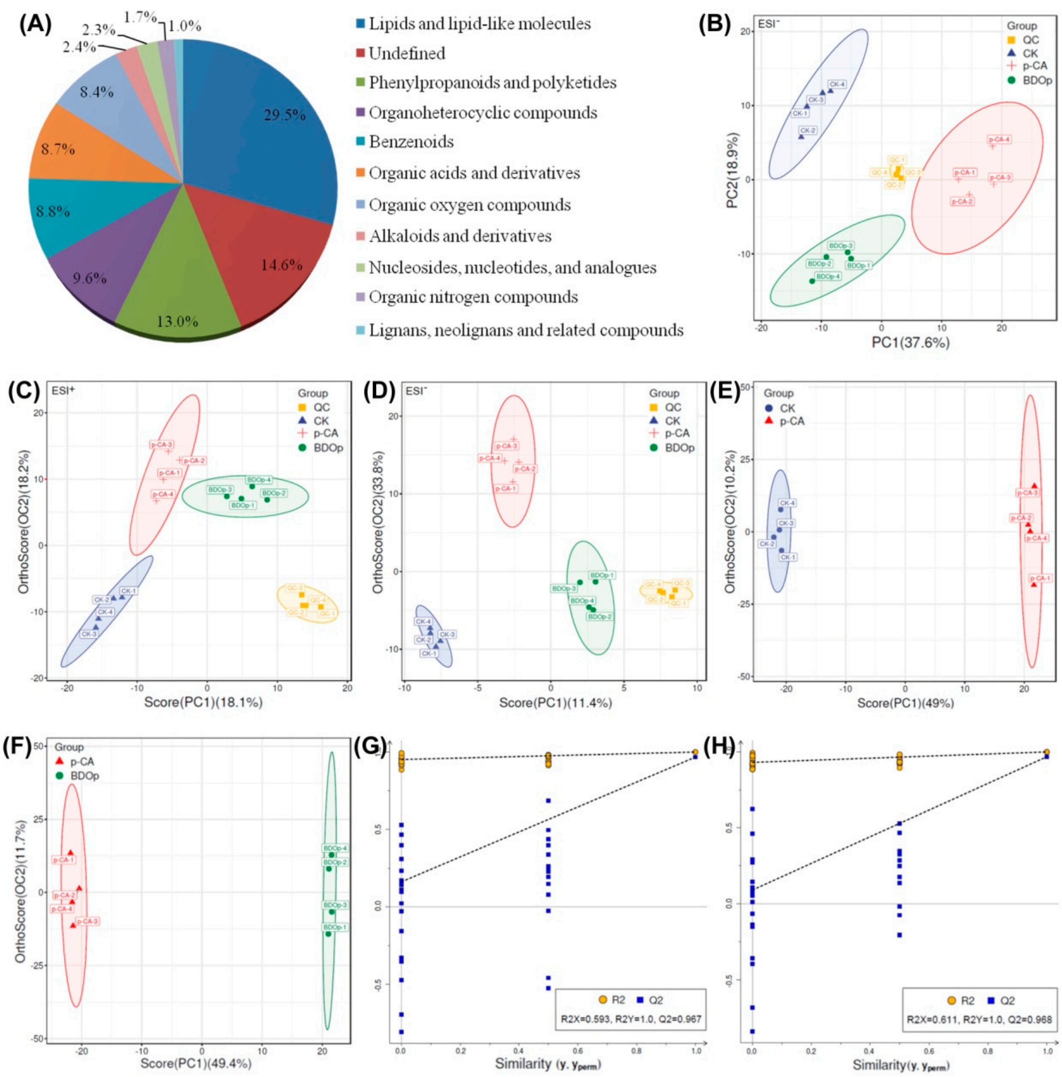


**Figure S2.** Pie chart of metabolite categorization (**A**). PCA score plot of metabolites under negative (ESI⁻) ion mode (**B**). OPLS-DA plots of metabolites in three different treatment groups under positive (ESI⁺) **(C)** and negative (ESI⁻) **(D)** ion mode. OPLS–DA score plot of metabolites in CK and *p*–CA group (**E**) and *p*–CA and BDOp group (**F**). Permutation test of OPLS–DA for CK vs. *p*–CA **(G)** and *p*–CA vs. BDOp **(H)**. (QC: quality control; CK: ddH$_2$O control group; *p*–CA: treatment group with 10 mL 400 mg/L *p*–coumaric acid; BDOp: *p*–coumaric acid with biochar–dual oxidant particles. Each treatment was subjected to four biological replicates)

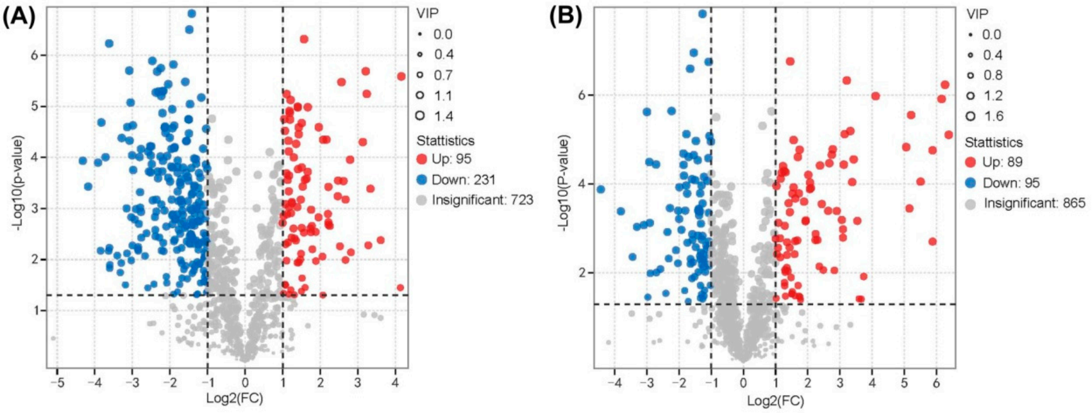


**Figure S3.** Volcano map of metabolites in *p*–CA vs. BDOp **(A)** and CK vs. BDOp group **(B)**.

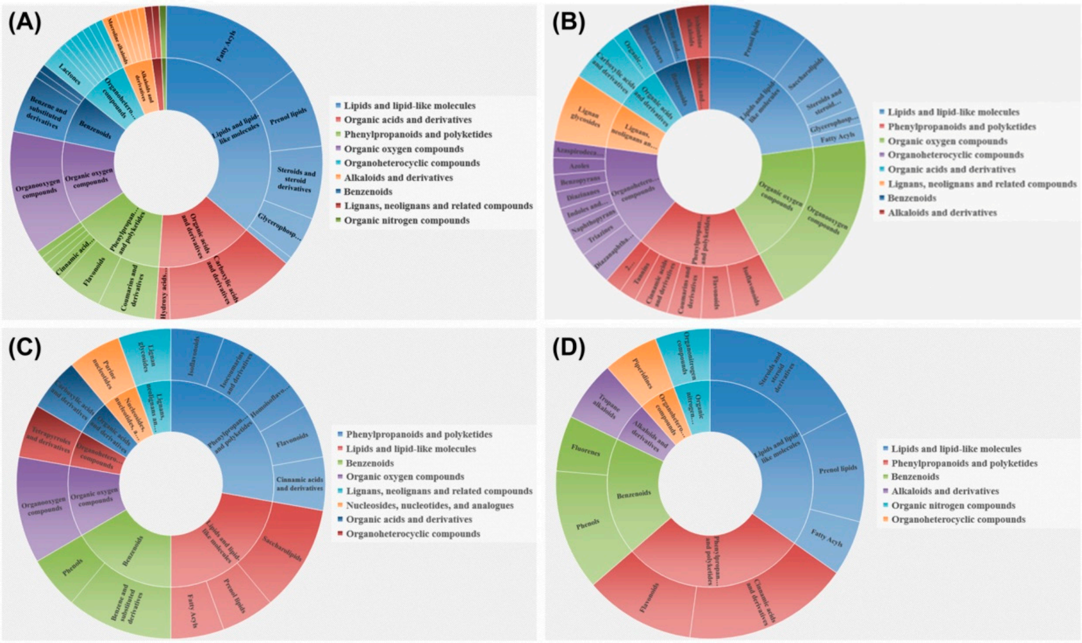


**Figure S4.** The sunburst chart for classification of the 165 up−regulated (**A**) and 59 down−regulated (**B**) DAMs in response to the *p*−CA stress, and 19 up−regulated (**C**) and 25 down−regulated (**D**) DAMs specifically regulated by the BDO composite particles.

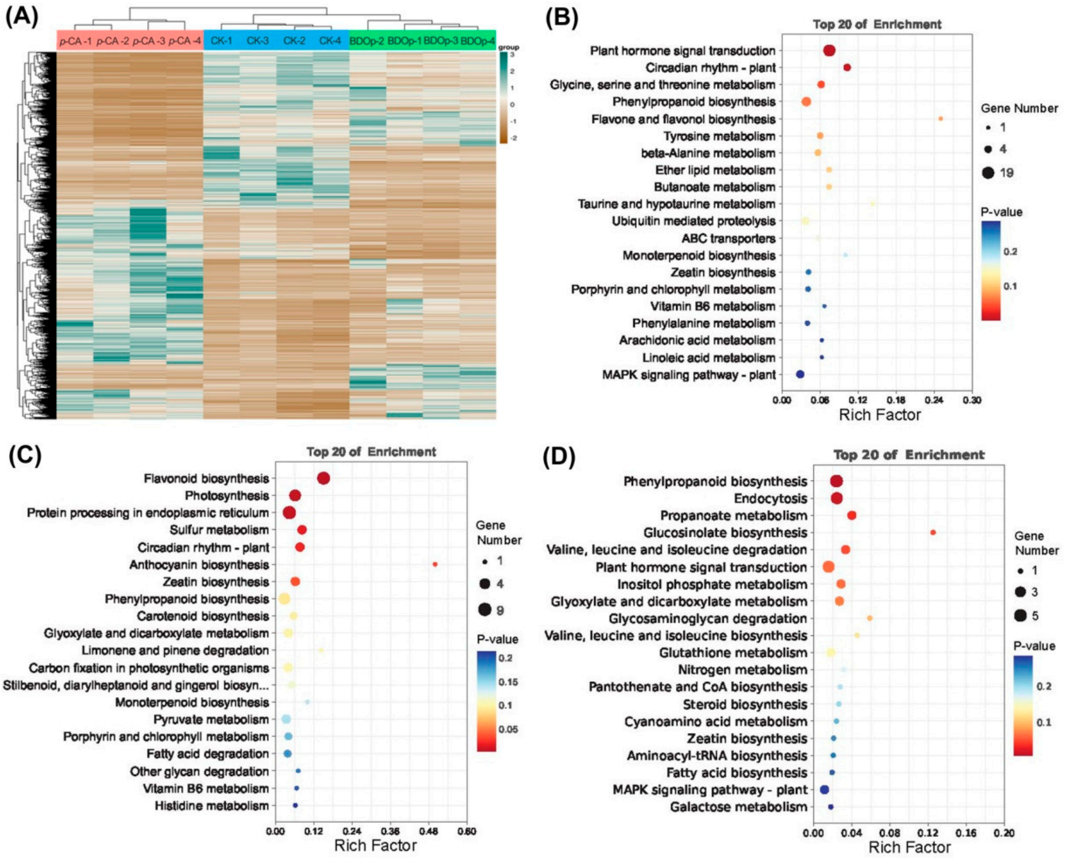


**Figure S5.** Hierarchical clustering heatmap of FPKM profiles in three treatments groups **(A)**. The bubble diagrams for the top 20 KEGG pathways for 863 down−regulated DEGs in response to the *p*−CA stress **(B)**, and 159 up−regulated **(C)** and 259 down−regulated **(D)** DEGs specifically regulated by the BDO composite particles. (CK: dd$H_2O$ control group; *p*−CA: treatment group with 10 mL 400 mg/L *p*−coumaric acid; BDOp: *p*−coumaric acid with biochar−dual oxidant particles. Each treatment was subjected to four biological replicates).

**Table S1.** Effect of different *p*–coumaric acid concentrations on the germination of tomato seeds.

| Concentration (mg/L) | GP (%) | GR (%) | RL (mm) |
|---|---|---|---|
| 0 | 86.5±1.9 a | 95.0±2.6 a | 61.7±1.9 a |
| 200 | 47.3±2.2 b | 93.0±3.5 a | 50.0±1.3 b |
| 400 | 0.0±0.0 c | 92.5±3.4 a | 23.9±1.2 c |
| 600 | 0.0±0.0 c | 84.0±4.9 b | 19.1±1.0 d |
| 800 | 0.0±0.0 c | 81.0±4.2 b | 8.5±0.4 e |
| 1000 | 0.0±0.0 c | 51.0±2.6 c | 4.6±0.2 f |

Abbreviations: GP, germination potential; GR, germination rate; RL, radical length. Each data presents the mean ± standard deviation ($n$ = 4). Different letters indicate statistically significant differences between treatments (One-way ANOVA, LSD test, $P < 0.05$).

**Table S2.** Specific primers were used for q-PCR.

| **Primer** | **Sequence（5'-3'）** | **Product（bp）** |
|---|---|---|
| CYP73A-F | ACCATAGACTCCTAGCGACCA | |
| CYP73A-R | GTCTTGTCCATTCCCCGTGA | 188 |
| LOX1_5-F | GGTTGCCACGTCTGAGTTTG | |
| LOX1_5-R | GACGCGGTCTCTGGGATTAC | 127 |
| LOX2S-F | CAATGGGAAAGGCGAGAGGA | |
| LOX2S-R | AGCGCCTGGGATAAGGTTTC | 137 |
| LCAT3-F | GTGCCATGCAAAGGTTCCTG | |
| LCAT3-R | TGGGTGCATTCTGACTTCCA | 101 |
| FAD2-F | CCACGAGAGGTGCATGTTCA | |
| FAD2-R | CGAATCCGTCAGAAGCAGGA | 169 |
| GAPDH-F1 | AGCCACTCAGAAGACCGTTG | |
| GAPDH-R1 | AGGTCAACCACGGACACATC | 189 |

**Table S3.** Effects of various treatments on the tomato seed germination indices

| Treatment | GP (%) | GR (%) | RL (mm) | RSA ($cm^2$) | GI (%) | SE |
|---|---|---|---|---|---|---|
| *p*-CA + BC-CP$_P$ | 57.0±2.6 | 89.0±3.8 | 37.7±1.7 | 0.353±0.017 | 57.2±2.7 | -0.30±0.01 |
| *p*-CA + BC-PS$_P$ | 0.0±0.0 | 31.0±1.1 | 3.1±0.1 | 0.079±0.004 | 1.6±0.1 | -0.89±0.00 |
| *p*-CA + CP-PS$_P$ | 53.0±2.6 | 76.0±1.6 | 9.1±0.6 | 0.209±0.010 | 11.8±0.3 | -0.56±0.00 |

Abbreviations: *p*–CA + BC-CP$_P$, 10 mL 400 mg/L *p*–coumaric acid with 0.0277 g biochar and calcium peroxide composite particles; *p*–CA + BC-PS$_P$, 10 mL 400 mg/L *p*–coumaric acid with 0.1230 g biochar and potassium persulfate composite particles; *p*–CA + CP-PS$_P$, 10 mL 400 mg/L *p*–coumaric acid with 0.0125 g calcium peroxide and potassium persulfate composite particles; GP, germination potential; GR, germination rate; RL, radical length; RSA, radical surface area; GI, germination index; SE, synthetical allelopathic effect index. Each data presents the mean ± standard deviation ($n$ = 4). Different letters indicate statistically significant differences between treatments (One–way ANOVA, LSD test, $P < 0.05$).
